# INTEREST GROUP SESSION—MEASUREMENT, STATISTICS, AND RESEARCH DESIGN: ANALYSIS OF DEVELOPMENTAL AND COMPLEX SYSTEMS: UNDERSTANDING THE DYNAMICS OF AGING

**DOI:** 10.1093/geroni/igz038.1378

**Published:** 2019-11-08

**Authors:** Xiao Yang, Nilam Ram, Nilam Ram

**Affiliations:** Pennsylvania State University, University Park, Pennsylvania, United States

## Abstract

Aging is the product of numerous dynamic processes that span multiple domains of functioning (e.g., biological, psychological, social), multiple levels of analysis, and multiple time-scales. Scientific inquiry in many fields has benefited from articulation and analysis of complex systems. This symposium brings together a collection of papers that illustrate how dynamical systems modeling is contributing to both theory and understanding of aging. Yang and colleagues apply Boolean network approach to intensive longitudinal data to identify sequences of emotion and behavior that lead to a stable equilibrium, and suggest how that information can be used to design interventions that push individuals toward a healthier equilibrium. Rector and colleagues illustrate use of dynamic indicators and multiscale entropy measures as indicators of resilience and explain how those measures may be used in prediction of physical recovery. Brick highlights how sequence mining methods can be used to identify commonalities and differences in dynamic change, and how those patterns characterize and distinguish groups with respect to aging trajectories. Moulder and colleagues demonstrate how latent maximum Lyapunov exponents can be used to study sensitivity of individuals’ developmental trajectories to initial conditions. Boker and colleagues provide a general overview of how dynamic models, including an adaptive equilibrium regulation model, distinguish resilience to acute versus chronic stressors and patterns of regulation. Together these papers highlight the value complex system thinking can add to our understanding and optimization of aging.

